# Increased Incidence of Epilepsy in a Brain Bank Alzheimer's Disease Cohort and Its Association With TDP‐43 Pathology

**DOI:** 10.1111/nan.70034

**Published:** 2025-08-22

**Authors:** Federica Rocca, Jaimee Kennedy, Shamis Osman, Zita Reisz, Andrew King, Istvan Bodi, Safa Al‐Sarraj, Claire Troakes

**Affiliations:** ^1^ London Neurodegenerative Diseases Brain Bank, Department of Basic and Clinical Neuroscience, Institute of Psychiatry, Psychology & Neuroscience King's College London London UK; ^2^ Department of Clinical Neuropathology King's College Hospital NHS Trust London UK

**Keywords:** Alzheimer's disease, epilepsy, hippocampal sclerosis, seizures, TDP‐43

## Abstract

**Aims:**

Evidence suggests Alzheimer's disease (ad) patients are at increased risk of epilepsy and that seizure incidence is associated with faster cognitive decline. Previous studies indicate hyperphosphorylated tau may play a role in this disease association; however, abnormal TDP‐43 and α‐synuclein deposition have not been extensively examined.

**Methods:**

Clinical and neuropathological records of ad cases over a 5‐year period were retrieved from the London Neurodegenerative Diseases Brain Bank. The 114 cases were categorised into three groups: ad plus epilepsy, ad plus hippocampal sclerosis (HS) and AD only. Semi‐quantitative scores for tau, TDP‐43 and α‐synuclein pathology within the middle temporal gyrus, hippocampus and amygdala were compared between groups.

**Results:**

A 12% incidence of epilepsy and/or epileptic symptomology was found among the cohort. Twelve cases (11%) showed HS. No significant difference in tau pathology scores was seen between groups. However, a significantly higher score for TDP‐43 was seen in ad plus epilepsy compared with ad only in the middle temporal gyrus (*p* = 0.004). The burden of α‐synuclein pathology was increased in the amygdala of ad plus epilepsy and ad plus HS.

**Conclusions:**

The incidence of epilepsy within this ad cohort is higher than expected within the general population (even when matched for age), and this may be associated with increased TDP‐43 burden. Understanding the relationship between ad and epilepsy may highlight mechanisms of cellular damage and tissue vulnerability.

## Summary

- Alzheimer's disease and epilepsy have been observed to occur, specifically in a cohort of Alzheimer's disease donors; 12% were found to have been given a clinical epilepsy diagnosis in life.
- Pathological assessment revealed a higher pathological score for TDP‐43 in the middle temporal gyrus of donors with coexisting Alzheimer's disease and epilepsy.
- A great majority of Alzheimer's disease donors developed epilepsy after Alzheimer's disease, suggesting that the latter may be a risk factor for epilepsy.

## Graphical abstract

Alzheimer's disease (AD) patients appear to show an increased risk of epilepsy. Clinical and neuropathological records of 114 AD cases were categorised into AD plus epilepsy, AD plus hippocampal sclerosis and AD only. A 12% incidence of epilepsy was found, higher than expected in the general population. Tau, TDP‐43 and α‐synuclein pathology were assessed. Compared to AD only, AD plus epilepsy showed higher TDP‐43 in the middle temporal gyrus and the burden of α‐synuclein pathology was increased in the amygdala.

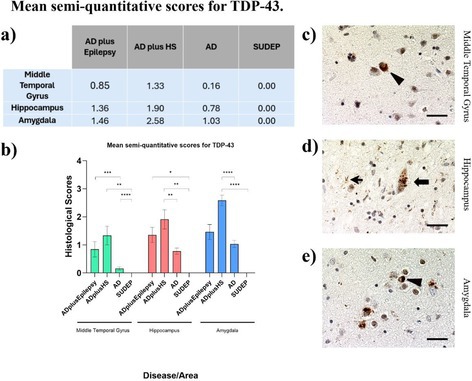

**Abbreviations**
AD: Alzheimer's disease
ASDs: antiseizure drugs
HS: hippocampal sclerosis
LBs: Lewy Bodies
LNts: Lewy neurites
MTG: middle temporal gyrus
SUDEP: sudden unexpected death in epilepsy
TDP‐43: transactive response DNA‐binding protein with 43 kDa

## 1 Introduction

Epilepsy is diagnosed in approximately 50 million people each year, with a higher prevalence in infants and older adults [1, 2]. Within the general world population, there is an incidence of 49 per 100,000 people in high‐income countries and an incidence of 139 per 100,000 people in middle‐ to low‐income countries [2]. Specifically, in England, there is an incidence of 14 in 1000 for adults aged between 80 and 84 [3]. In older adults, the most common epilepsy types, in cases of new‐onset epilepsy, are induced by the presence of tumours, infections or cerebrovascular disease [4].

In 2017, the International League Against Epilepsy (ILAE) established criteria for diagnosing epilepsy, which state that a diagnosis can be made when a patient suffers one unprovoked (or reflex) seizure and has a high probability (60%) of suffering from an additional unprovoked seizure, or if the patient suffered two unprovoked (or reflex) seizures at least 24 h apart [5]. However, many Alzheimer's disease (ad) patients may suffer from sporadic and/or nonconvulsive seizures and never receive a clinical diagnosis of epilepsy [6].

Several research studies have found an association between epilepsy and ad [7, 8]. Observational research carried out using the National Alzheimer's Coordinating Centre database from the University of California revealed an association between seizures and increased likelihood of being diagnosed with ad and also an increased prevalence of seizures in people already diagnosed with ad, concluding that ad patients had an increased probability of presenting with seizures after a diagnosis of ad [9]. Evidence of seizure activity is commonly seen in patients with early‐onset ad (EOad), where cognitive symptoms appear before 65 years old, with an incidence of epilepsy of 40% observed in one cohort, specifically 3–5 years after EOad diagnosis [10]. Additionally, patients suffering from both diseases showed longer duration of ad symptoms and worse performance in cognitive tests compared with EOad patients with no epilepsy.

Similarly, research focused on temporal lobe epilepsy (TLE) has demonstrated that uncontrolled seizures have a detrimental effect on cognition, and neuropathological changes comparable with those observed in ad patients may be observed in TLE. In particular, increased expression of βAPP along with upregulation of tau was observed in the hippocampus of TLE patients who underwent temporal lobe resection [11, 12]. The involvement of tau in seizure activity has also been observed in animal models. Inducing status epilepticus in male mice revealed the presence of hyperphosphorylated tau in the mice hippocampi in epileptogenic and seizure propagating areas through time, demonstrating evidence of the expression of similar pathological markers as those observed in ad [13].

ad is often comorbid with limbic Lewy Body (LB) pathology (characterised by the presence of pathological α‐synuclein in the form of LBs or Lewy neurites [LNts]), which could influence the progression of ad pathology [14, 15]. Interestingly, elevated aggregation of α‐synuclein has been found in the serum and cerebrospinal fluid of patients with intractable epilepsy [16]. Furthermore, in cases of ad and Lewy Body Dementia, the incidence of developing seizures and myoclonus is higher when the two pathologies coexist [14].

Some ad patients also have transactive response DNA‐binding protein of 43 kDa (TDP‐43) inclusions on histology, with phosphorylated TDP‐43 pathology being shown in approximately 57% of ad patients [17] and limbic age‐related TDP‐43 encephalopathy (LATE‐NC) found in around 50% of ad patients [18]. In human tissue, TDP‐43 has been observed to colocalise with hyperphosphorylated tau and amyloid‐β, and in animal models of AD, it was found to impact disease progression by decreasing the expression of calcineurin, which is inversely associated with Braak stages [19]. Phosphorylated TDP‐43 is also seen in cases of hippocampal sclerosis (HS), which can develop due to neurodegeneration, ageing or due to chronic epileptic changes [20, 21]. Although there has been some previous investigation into the role of tau and α‐synuclein in epilepsy and seizure activity, there is little research focused on the involvement of TDP‐43 [11, 22]. The present observational study aimed to assess the incidence of epilepsy in a Brain Bank cohort of ad donors and to investigate potential differences in the pathological load when the two conditions coexist compared to individually, focusing on tau, TDP‐43 and α‐synuclein.

## 2 Methods

### 2.1 Data Collection

**FIGURE 1:**
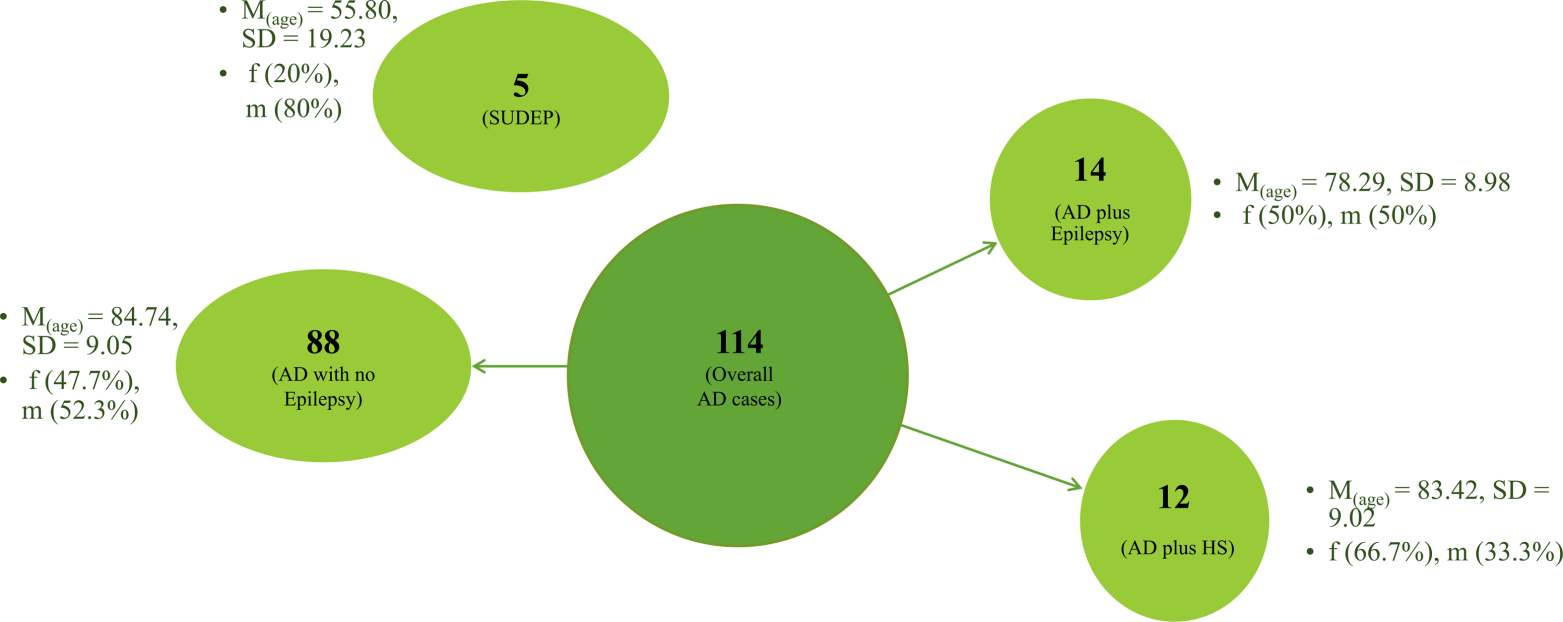
Schematic representation of the five cohorts and their demographic information. The sample consisted of 114 donors with ad, divided into ad plus epilepsy (received a clinical epilepsy diagnosis or had a history of seizure activity) and ad plus HS (evidence of HS at neuropathological examination, without clinical epilepsy diagnosis or history of seizures), and five SUDEP donors. Two cases of ad plus epilepsy also showed HS, these cases were included within the ad plus epilepsy group only.

The London Neurodegenerative Diseases Brain Bank (King's College London) archives were searched to identify all brain donors from a 5‐year time period (2017–2022) with a neuropathological diagnosis of ad (modified Braak/BNE Stage III and above [23, 24]). From this total cohort of 114 donors, the clinical records and neuropathological reports were mined for evidence of epilepsy, or of HS (Type 1 presents neuronal loss and gliosis in CA1 and CA4 segments; Type 2 is characterised by neuronal loss and gliosis in CA1 segment only, which is specifically found in cases of TLE with HS; and Type 3 where neuronal loss and gliosis are mainly present in CA4 only [25]). Additionally, a group of five donors who died from sudden unexpected death in epilepsy (SUDEP) was selected, amounting to 119 cases assessed in total. Donors were divided into four groups: ad only (ad donors with no recorded history of epilepsy or HS), ad plus epilepsy (ad donors with evidence of epilepsy), ad plus HS (ad donors solely with neuropathological evidence of HS, without a clinical diagnosis of epilepsy or history of seizures) and SUDEP (Figure 1). For those that had both ad and epilepsy, information on the time of onset of epilepsy compared with ad onset was recorded. Information on AD duration and whether the donors were prescribed antiseizure drugs (ASDs) during their lifetime was also gathered across cohorts.

### 2.2 Immunohistochemistry

Immunohistochemistry and semi‐quantitative assessment were performed as part of the original diagnostic process or, where analysis of hyperphosphorylated tau (AT8), phosphorylated TDP‐43 or α‐synuclein had not been carried out in any brain area, conducted as part of this project. In both circumstances, the tissue examined was 7‐μm‐thick formalin‐fixed paraffin‐embedded sections of middle temporal gyrus, hippocampus and amygdala, to assess the AD pathology, which typically spreads to the limbic areas from Braak/BNE Stage III [23, 24]. Immunostaining was performed using an antibody against tau‐targeting antibody AT8 (MN1020, Invitrogen), against phosphorylated TDP‐43 (TIP‐PTD‐M01A, Cosmo Bio Ltd) and against alpha‐synuclein (8060521, Insight Biotechnology, 610786, BD Transduction Labs). The procedure was carried out on the Leica Bond platform at the Clinical Neuropathology Department of King's College Hospital. To visualise stains, the polymer detection kit (Leica DS9800) was used, and sections were counterstained with haematoxylin.

### 2.3 Assessment of Pathology

Protein aggregation and localisation for hyperphosphorylated (HP‐)tau, pTDP‐43 and α‐synuclein, within the areas of interest for each case, were assessed and scored using a semi‐quantitative scale. In summary, HP‐tau thread and neuritic plaque deposition was assessed at lower power (objective ×20, eyepieces ×10) and calculated depending on whether there was no deposition (absent/0), only very occasional difficult to detect deposition (mild/1), easily detectable but not in every relevant field (moderate/2) or seen in abundance in every relevant field (severe/3). The whole blocks were examined in this fashion. The approximate whole block areas (grey and white matter) were posterior hippocampus (60 mm^2^), amygdala (120 mm^2^) and middle temporal gyrus (200 mm^2^). Semi‐quantitative scores were also obtained for HP‐tau positive neurofibrillary tangles, and TDP‐43 positive neuronal and glial cytoplasmic inclusions, and α‐synuclein positive LBs and Nts. These stains were assessed under high power (objective ×40, eyepieces ×10), and again, the whole blocks were examined as to whether there was no deposition (absent/0), only very occasional difficult to detect deposition (mild/1), easily detectable in most but not every relevant field (moderate/2) and seen in every or nearly every relevant field (severe/3). For most cases, this had been carried out during the original neuropathological diagnosis and scores were mined from the neuropathological reports. For any missing data, new immunohistochemical stains were carried out and sections assessed by a neuropathologist (I Bodi) in exactly the same manner.

### 2.4 Statistical Analysis

Statistical analysis was conducted using SPSS version 29. Descriptive statistics analysis was carried out across all groups to investigate the incidence of occurrence of epilepsy in an ad cohort and identify possible differences across sexes. To assess differences in pathological load, the means of the pathological scoring given to donors for neuritic plaques, tangles, neuropil threads and glial/astrocytic tau, TDP‐43 inclusions and LBs and LNts were calculated. A Kruskal–Wallis test with Dunn's post hoc test and Bonferroni correction analysis was performed to assess which of the four cohorts had higher scoring for tau neuritic plaques, tangles, neuropil threads and glial/astrocytic tau, for TDP‐43 inclusions, and higher scoring for LBs and LNts.

## 3 Results

### 3.1 Incidence of Epilepsy in an ad Cohort

This study revealed that, within a total of 114 donors diagnosed with ad (Braak, BNE Stage III or above), 14 (12%) showed epileptic symptomology. The prevalence of epilepsy within the ad donors (*M*_(age)_ = 78.29, SD = 8.98) is higher than the reported incidence in the general population (0.94%) [3]. Additionally, 12 (11%) of the overall cohort had no seizures or clinical diagnosis of epilepsy but showed pathological findings of HS (CA1 and subiculum predominant; see Table 1) (ad plus HS) (*M*_(age)_ = 83.42, SD = 9.02). The remaining 88 donors (77%) were grouped in the ad‐only cohort (*M*_(age)_ = 84.74, SD = 9.05) (Figure 1).

**TABLE 1:** Comorbidities in a brain bank AD cohort (E_n_ = donor in the AD plus epilepsy group, HS_n_ = donor in the AD plus HS group).

Donors	Age	Sex	HS	TDP‐43 pathology^a^, ^b^	LB pathology^c^
E_1_	65	M		Josephs' Stage 6 (LATE‐NC Stage 3)	Limbic predominant (McKeith criteria)
E_2_	74	F			
E_3_	78	F		Josephs' Stage 3	
E_4_	75	M	CA1 segment and Subiculum	Josephs' Stage 5	Amygdala predominant (McKeith criteria)
E_5_	93	M			
E_6_	65	M		Josephs' Stage 4	Amygdala predominant (McKeith criteria)
E_7_	73	M		Josephs' Stage 4	Limbic predominant (McKeith criteria)
E_8_	75	M		Josephs' Stage 2 (LATE‐NC Stage 2)	
E_9_	91	F		Josephs' Stage 6 (LATE‐NC Stage 3)	
E_10_	76	M		Josephs' Stage 2	Limbic predominant (McKeith criteria)
E_11_	92	F		Josephs' Stage 4	Limbic predominant (McKeith criteria)
E_12_	85	F		Josephs' Stage 2	Amygdala predominant (McKeith criteria)
E_13_	79	F		Josephs' Stage 1	Limbic predominant (McKeith criteria)
E_14_	75	F	CA1 segment and Subiculum	Josephs' Stage 3	
HS_1_	64	F	CA1 segment and Subiculum	Josephs' Stage 4 (LATE‐NC Stage 3)	
HS_2_	94	M	CA1 segment and Subiculum	LATE‐NC Stage 3	
HS_3_	75	F	CA1 segment and Subiculum	Josephs' Stage 6 (LATE‐NC Stage 3)	Limbic predominant (McKeith criteria)
HS_4_	91	M	CA1 segment and Subiculum	Josephs' Stage 3 (LATE‐NC Stage 2)	Neocortex predominant (McKeith criteria)
HS_5_	87	F	CA1 segment and Subiculum	Josephs' Stage 3 (LATE‐NC Stage 2)	Amygdala predominant (McKeith criteria)
HS_6_	80	F	CA1 segment and Subiculum	Josephs' Stage 1 (LATE‐NC Stage 1)	
HS_7_	84	M	CA1 segment and Subiculum	Josephs' Stage 4	
HS_8_	77	F	CA1 segment and Subiculum	Josephs' Stage 2	
HS_9_	79	M	CA1 segment and Subiculum	Josephs' Stage 6	
HS_10_	91	F	CA1 segment and Subiculum	Josephs' Stage 5	Neocortex predominant (McKeith criteria)
HS_11_	84	F	CA1 segment and Subiculum	Josephs' Stage 4	
HS_12_	95	F	CA1 segment and Subiculum	Josephs' Stage 6	

^a^ TDP‐43 pathology in AD, staging scheme according to Joseph's staging scheme [26].

^b^ LATE‐NC staging scheme based on the anatomical spreading of TDP‐43 pathology [27].

^c^ Lewy Body staging scheme according to the Lewy pathology, McKeith criteria [28].

The SUDEP cohort assessed was slightly younger compared with other groups (*M*_(age)_ = 55.80, SD = 19.23). Two cases, grouped in ad plus epilepsy, also demonstrated the presence of HS at post‐mortem examination. In these cases, HS was neuropathologically suggested to have developed due to neurodegenerative changes, since neuronal loss was observed in the CA1 segment and subiculum; therefore, for analysis, they were still included in their original clinically based group (Figure 1 and Table 1). Through an observational analysis of clinical records, it was found that in the ad plus epilepsy cohort, 71.4% of donors developed epilepsy after ad diagnosis, whereas 28.6% had epilepsy before being diagnosed with ad. Furthermore, it was found that ASDs had been prescribed to 79% of the ad plus epilepsy donors, 33.3% of the ad plus HS donors and 11.4% of the ad‐only donors. Information on ad duration was investigated, revealing ad plus epilepsy had the longest disease duration (*M*_(years)_ = 12.36, SD = 6.39), followed by ad plus HS (*M*_(years)_ = 9, SD = 4.12), and ad‐only donors showing the shortest disease duration (*M*_(years)_ = 7.06, SD = 3.40). Analysis also revealed a 9.7% incidence of EOad in the ad cohort, an incidence of 28.6% in ad plus epilepsy and a 16.7% incidence in ad plus HS. Different pathologies were found to co‐occur along with ad; in particular, donor E_4_ (Table 1), in the ad plus epilepsy group, was diagnosed with ad, epilepsy, HS, TDP‐43 pathology and LB pathology.

### 3.2 Neuropathological Staging for Tau

**FIGURE 2:**
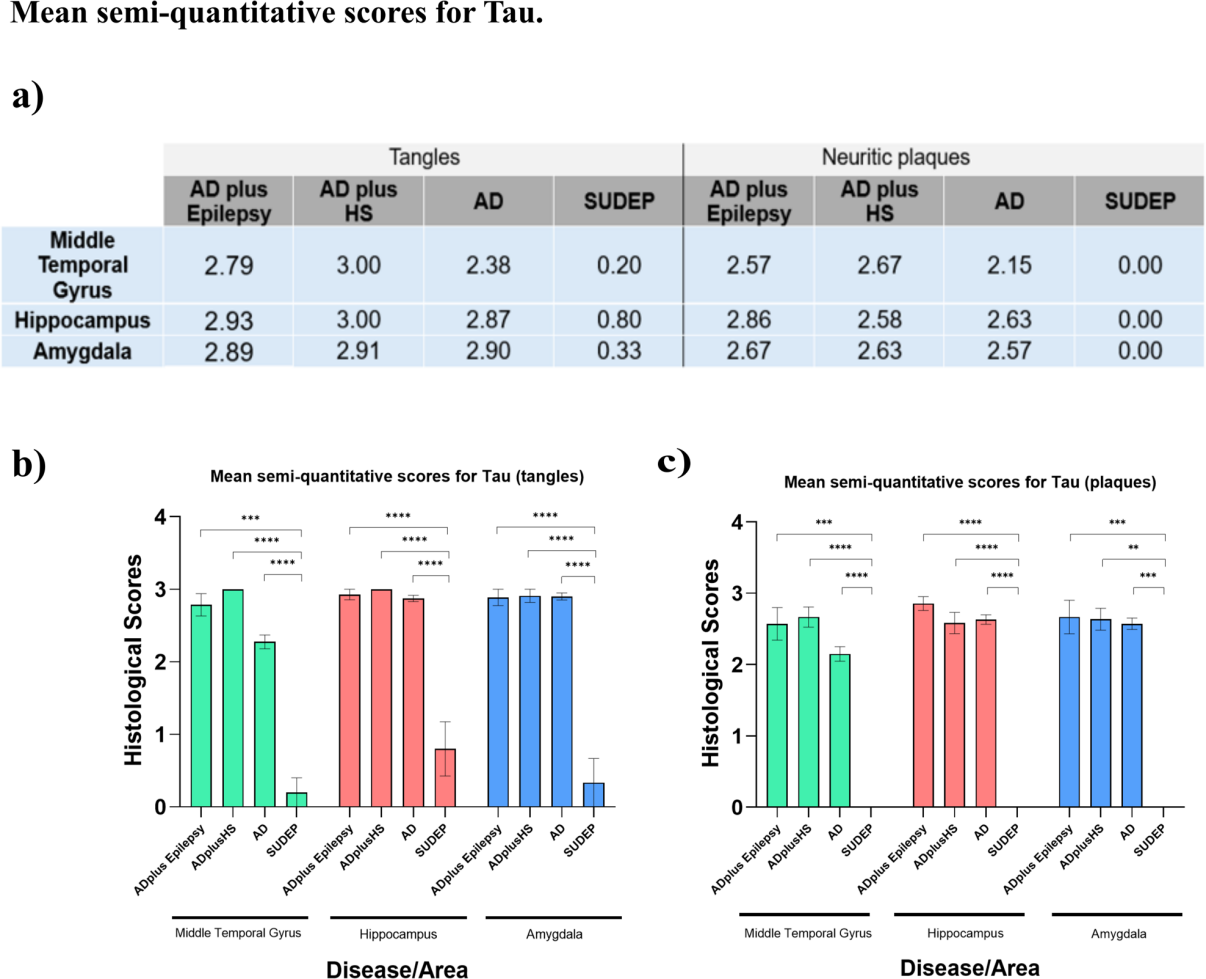
Semiquantitative scoring for tau inclusions. (a) Mean semi‐quantitative scores for tau‐positive tangles and plaques within the MTG, hippocampus and amygdala. There was no significant difference found between the ad epilepsy cohort and the ad‐only group for tau tangles (b) or plaques (c) in any of the regions examined. The majority of groups/regions displayed a significantly higher mean tau score for both tangles and plaques in comparison with epilepsy‐only donors (*p < 0.05, **p < 0.01, ***p < 0.005, ****p < 0.001). Bars show mean semi‐quantitative protein scores (± SEM).

As expected, all ad donors showed high mean scores for tau pathology (both tangles and neuritic plaques) across all three regions. No significant difference was found between the three ad groups. Also, as expected, SUDEP cases showed low scoring for both tau tangles and tau neuritic plaques across brain regions, which was significantly different from the other four groups (Dunn's post hoc test with Bonferroni correction) (Figure 2a).

### 3.3 Neuropathological Staging for TDP‐43

**FIGURE 3:**
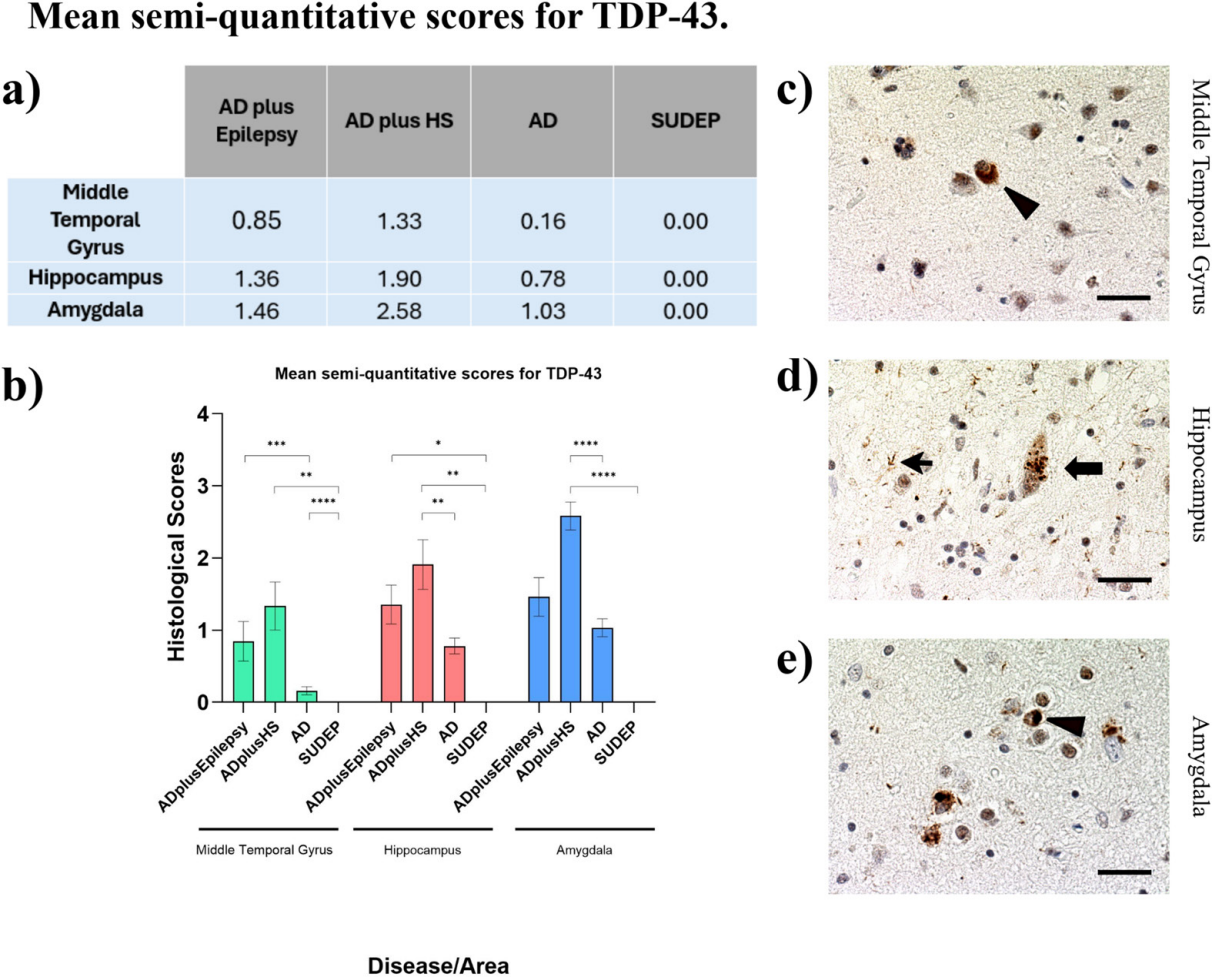
Semi‐quantitative scoring for TDP‐43 inclusions. (a) Mean semi‐quantitative scores for TDP‐43 immunopositivity within each group and region. (b) Representative images of TDP‐43 pathology within an ad plus HS case. (c, d, and e) A significantly higher mean TDP‐43 score was found in the ad plus HS group (in all regions) and in the ad plus epilepsy (in MTG) compared with ad alone (*p < 0.0.05, **p < 0.01, ***p < 0.005). Bars show mean semi‐quantitative protein scores (± SEM). Scale bar: 20 μm.

The calculated mean scores (Figure 3a) showed that in all three areas, ad plus HS had the highest score for TDP‐43 inclusions and that this was significantly higher than in ad only. ad plus epilepsy was found to have higher TDP‐43 scores compared with ad only. In the middle temporal gyrus, there was a statistically significant difference between SUDEP and ad plus HS (*p* = 0.009, Dunn's post hoc test, with Bonferroni correction) and ad only and ad plus epilepsy (*p* = 0.004). In the hippocampus, a significant difference was observed between epilepsy and ad plus epilepsy (*p* = 0.04), epilepsy and ad plus HS (*p* = 0.006) and between ad and ad plus HS (*p* = 0.01). In the amygdala, a significant difference was observed between epilepsy and ad plus HS (*p* < 0.001); donors in ad plus epilepsy were found to have the second highest mean rank for pathological load; however, *p* = 0.05 suggests a 5% possibility that the result may be due to chance.

### 3.4 Neuropathological Staging for α‐Synuclein

**FIGURE 4:**
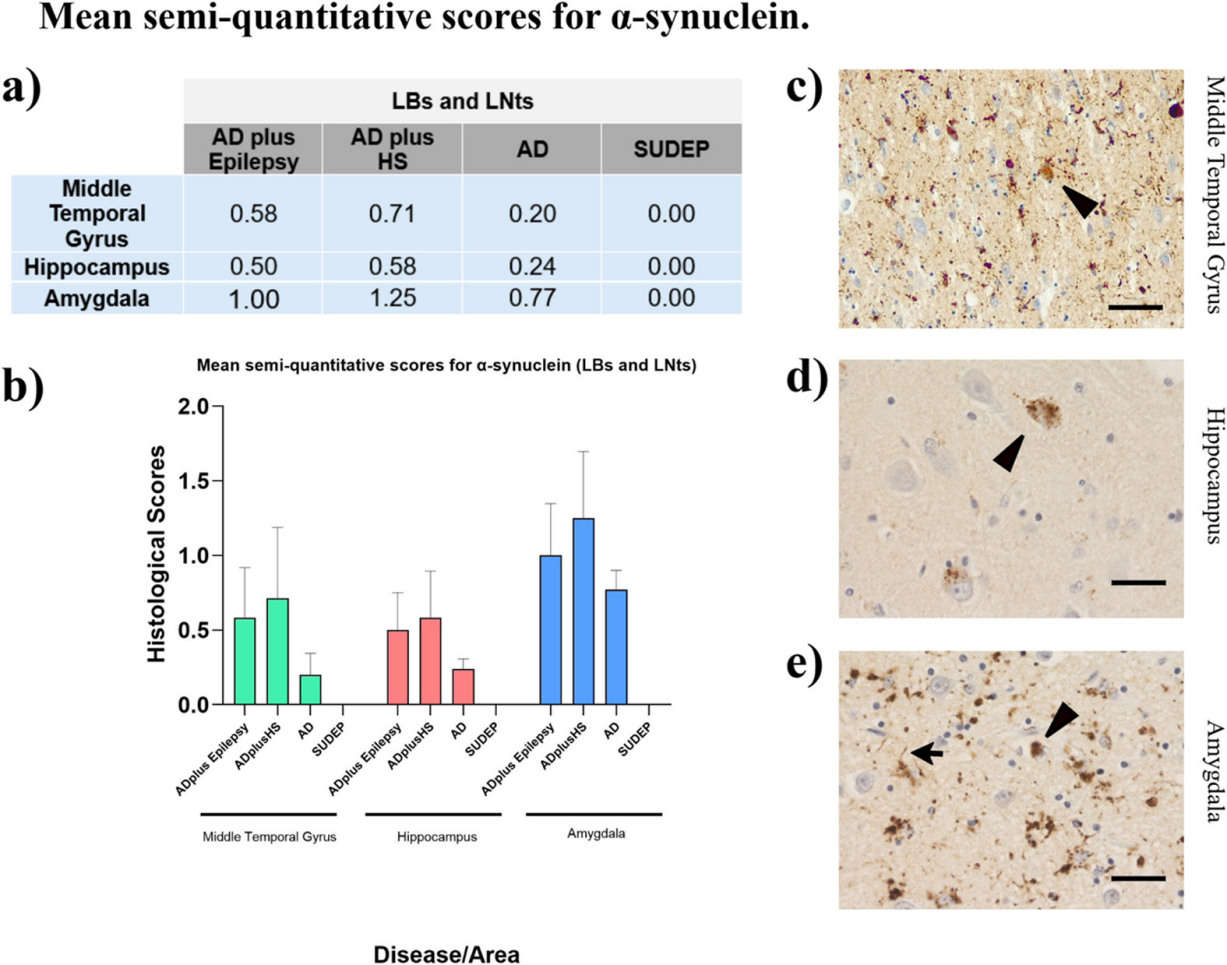
Semi‐quantitative scoring for α‐synuclein. (a) Mean semi‐quantitative scores for alpha‐synuclein positivity in the form of Lewy Bodies. Although high scores were seen in some ad with epilepsy cases, there was no significant difference between pathology scores in any groups or regions for either Lewy Bodies (b). Bars show mean semi‐quantitative protein scores (± SEM). (c) Histological evidence of α‐synuclein inclusions in donors with AD plus epilepsy, and (d and e) histological evidence of α‐synuclein inclusions in donors with AD plus HS, showing Lewy Bodies (black arrowhead) and dystrophic neurites (black arrow). A small, not statistically significant difference was found among donors for α‐synuclein pathological load. Scale bar: 20 μm.

Mean scores were calculated for α‐synuclein LBs and LNts, showing that most groups were given low scores for inclusions (Figure 4); however, in the middle temporal gyrus, hippocampus and amygdala, the highest mean ranks were found for ad plus HS donors. ad plus epilepsy had higher mean ranks compared with ad‐only donors across all areas (Figure 4a). Although small differences can be observed across cohorts, analysis did not find any statistically significant differences in LB and LNt inclusions in the middle temporal gyrus (*p* = 0.49), hippocampus (*p* = 0.40) and amygdala (*p* = 0.32).

## 4 Discussion

The aim of the present study was to explore the incidence of epilepsy in a cohort of ad patients, using the London Neurodegenerative Diseases Brain Bank archives, and to examine the difference in pathological load for tau, TDP‐43 and α‐synuclein within ad cases with and without epilepsy. The results showed a 12% incidence of epilepsy, considerably higher than the 0.94% prevalence reported in the general population [2]. An important finding was that most of the donors in ad plus epilepsy developed the disorder after the diagnosis of ad, which is in keeping with findings showing that ad patients have a higher likelihood of developing seizure activity [9]. Four of the ad plus epilepsy donors developed epilepsy before ad, two of them had their first seizure within a month of their ad diagnosis, suggesting that these may be a sign of brain vulnerability due to ad. Another interesting result was that ad duration for ad plus epilepsy donors was extremely long (up to 30 years in one case); previous research has found that the prevalence of seizures in patients suffering from dementia increase of 0.64% each year of disease duration [29], representing an additional variable that may influence the association between seizure activity and ad.

Although the present sample included only a few donors with EOad, it is important to note that 14.2% of ad plus epilepsy donors were among them; as observed in other research, there is a high incidence of epilepsy in EOad patients [10]. Moreover, a Mendelian randomisation study found that genetic predisposition to ad is associated with a higher risk of developing generalised epilepsy [30]. The present database analysis revealed that a small percentage (21.4%) of ad plus epilepsy were not prescribed ASDs, which could be due to nonconvulsive seizure aetiology that has been observed in some ad patients diagnosed with epilepsy [6]. It was also discovered that 13% of ad‐only donors were prescribed ASDs during the course of their disease. Even though this is an interesting finding, it is also important to mention that ASDs, such as benzodiazepines, are often prescribed for ad patients with neuropsychiatric symptoms [31]. Nevertheless, their influence in altering possible epileptic manifestations needs to be considered.

As expected, semi‐quantitative scoring for tau was high for all ad groups, likely due to the advanced ad stages (Braak Stage III or over) where tau inclusions are extensively present and diffused throughout the limbic areas [23]. SUDEP donors were found to have significantly lower pathological load than other donors for tau inclusions, which could be associated with the younger age of the donors.

TDP‐43 pathological scoring was found to be the highest in ad plus HS in all limbic regions, which was an expected outcome since previous research shows that TDP‐43 inclusions can be found in HS [32]. Typically, HS ageing is associated with the presence of TDP‐43 inclusions in the hippocampus. HS can also be categorised into HS ageing and HS due to neurodegeneration, which are characterised by loss of pyramidal neurons and gliosis primarily in the CA1 segment and subiculum [25]. The results show that ad plus epilepsy donors had higher TDP‐43 scores in the middle temporal gyrus, hippocampus and amygdala compared with ad‐only donors. Although previous studies have reported that TDP‐43 is not involved in the pathology of epilepsy [33], it was found that pathological TDP‐43 inclusions aggravate the pathological burden. Particularly, in co‐occurrence with HS, it was linked to worse cognitive performance and in a cognitive normal ageing cohort to a 75% probability of developing ad later in life [21]. Interestingly, in the ad plus epilepsy group, 11 out of 14 donors had TDP‐43 pathology, six of whom were at a severe stage, three had LATE‐NC, whereas six out of 12 donors with ad and HS were found to have LATE‐NC, and the other six had TDP‐43 pathology (Table 1).

The present results support the possibility that TDP‐43 interacts with the pathological hallmarks of ad and leads to further damage. Both pathological tau and TDP‐43 are found to colocalise and interact in the hippocampus of ad donors [34]. The presence of pathological TDP‐43 was found to interact with pathological tau, worsening tau burden in cases of ad comorbid with LATE‐NC, increasing pathological tau seeding potential [35]. Their interaction was also observed in animal models, such as the evidence gathered from a study on *C. elegans* , which has observed that TDP‐43 promotes tau aggregation and neurotoxicity [36]. In particular, it was found that in transgenic animals presenting comorbid tau and TDP‐43, there was an increased loss of GABA‐ergic neurons, which was consistent throughout ageing. Transmission of GABA is disrupted in several epilepsy types, such as generalised epilepsy [37], and GABA receptor expression was observed to be negatively associated with cell damage and cell death in the amygdala of patients with MTLE [38]. This may suggest that the interaction of TDP‐43 and pathological tau may be involved in ad plus epilepsy, where a higher pathological load of TDP‐43 was found compared with the pathologies on their own.

Although α‐synuclein inclusion scores were not statistically different across the five groups, likely due to limbic LB pathology being a very common coexisting pathology in ad [14], interestingly, the pathological load of α‐synuclein inclusions revealed some increases that can be observed in the mean scoring for ad plus epilepsy in the amygdala. This is in keeping with previous literature showing that α‐synuclein presence is associated with higher seizure risk [14]. TDP‐43 was also found to interact with α‐synuclein. It has been observed that TDP‐43 can create fibrils of TDP‐43 and α‐synuclein together, by binding with several α‐synuclein species; furthermore, by coincubating cells of human neuroblastoma containing these fibrils, higher neurotoxicity was found compared with cells incubated with only TDP‐43 fibrils or α‐synuclein fibrils [39]. Moreover, research conducted on abnormal TDP‐43 expression in brain donors suffering from ad, limbic ad and ad with LB pathology found colocalization of hyperphosphorylated tau and occasionally also α‐synuclein pathology, with TDP‐43 pathology in seven out of 12 cases, showing TDP‐43 positivity in amygdala and/or entorhinal cortex [33]. An association has been noted between the number of comorbidities and poor performance in cognitive tests, such as the MMSE, revealing that several co‐occurring pathologies lead to more severe cognitive impairment [40]. The findings from the present research suggest that the synergy of TDP‐43, tau and α‐synuclein may aggravate cell damage and tissue vulnerability and lead to an increased risk of developing seizure activity or epilepsy as the pathology progresses to more severe stages. Further research will be necessary to investigate the colocalization of the proteins and their potential interactions.

In conclusion, this study showed an increased incidence of epilepsy found among ad donors, compared with the incidence rate among the age‐matched general population in England. Although the observational analysis conducted here is necessary to investigate the incidence of co‐occurrence of the two pathologies, the information gathered was limited by the sample availability within the Brain Bank and the availability of clinical information provided per donor, such as information on seizure semiology and epidemiology, which was not present in the majority of the donors' medical records. Therefore, in future research, it will be important to assess a larger sample size, controlling for a more representative population. Moreover, because of standard brain banking preservation methods, evidence of HS was only investigated unilaterally [41]; thus, the presence of HS may be higher than the one reported in the present analysis. Nevertheless, these findings emphasise the importance of investigating protein interactions to better understand the molecular mechanisms characterising the comorbidity of ad and epilepsy, which may be associated with increased TDP‐43 pathology. The identification that a large number of the ad donors developed epilepsy after the diagnosis of ad suggests the potential for ad to be a risk factor for new‐onset epilepsy in older adults. Many ad patients who develop epilepsy later in life are often misdiagnosed because of the variety of seizure manifestations, particularly nonconvulsive seizures, where loss of awareness and confusion are often overlooked as symptoms related to ad [6]. Therefore, it is crucial to further explore the coexistence of ad and epilepsy to improve clinical practice and treatments for patients suffering from this comorbidity. Additionally, shedding light on how these proteins interact throughout the progression of the pathology is necessary for the development of possible therapeutic interventions.

## Author Contributions

Study conception and design were carried out by J.K. and C.T. Material preparation was performed by F.R., J.K., S.O. and C.T. Data analysis was conducted by F.R., Z.R., A.K., I.B. and S.A.S. All authors worked on the manuscript preparation and read and approved the final manuscript.

## Ethics Statement

Informed consent was given by all donors and/or relatives of donors for the use of tissue in research. Ethical approval for the use of post‐mortem human tissue in this study falls under the London Neurodegenerative Diseases Brain Bank ethical approval (REC code 23/WA/0124).

## Conflicts of Interest

The authors declare no conflicts of interest.

## Supporting information


**Data S1:** Supporting information.

## Data Availability

The data that support the findings of this study are available from the corresponding author upon reasonable request.
